# Dental Management of Ectodermal Dysplasia: Two Clinical Case Reports

**DOI:** 10.5681/joddd.2012.023

**Published:** 2012-09-01

**Authors:** Somayeh Hekmatfar, Karim Jafari, Raziyeh Meshki, Samaneh Badakhsh

**Affiliations:** ^1^Post-graduate Student, Department of Pediatric Dentistry, Faculty of Dentistry, Shiraz University of Medical Sciences, Shiraz, Iran; ^2^Post-graduate Student, Department of Prosthodontics, Faculty of Dentistry, Shiraz University of Medical Sciences, Shiraz, Iran; ^3^Assistant Professor, Department of Pediatric Dentistry, Faculty of Dentistry, Ahwaz University of Medical Sciences, Ahwaz, Iran

**Keywords:** Anodontia, hypodontia, oligodontia, oral rehabilitation, removable prosthesis

## Abstract

Ectodermal dysplasia is a hereditary disorder associated with abnormal development of embryonic ectodermally-derived organs including teeth, nails, hair and sweat glands. Hypodontia of the primary and permanent dentition is the most com-mon oral finding. Therefore, affected patients need dental prosthetic treatments during their developmental years. This re-port presents two cases of children affected by ectodermal dysplasia with anodontia. Oral rehabilitation was accomplished with removable acrylic prostheses. Treatment had major impacts on self-esteem, masticatory function, speech and facial esthetic.

## Introduction

Hereditary ectodermal dysplasia represents a large group of conditions in which two or more ectodermally-derived anatomic structures fail to develop. Patients with ectodermal dysplasia are characterized by hypoplasia or aplasia of structures such as skin, hair, nails, teeth, nerve cells, sweat glands, parts of the eye and ear and other organs.^1^ Ectodermal dysplasia might be inherited in any form of several genetic patterns including autosomal-dominant, autosomal-recessive, and X-linked modes.^2^ Although more than 170 different subtypes of ectodermal dysplasia have been identified, these disorders are considered to be relatively rare with an estimated incidence of 1 case per 100,000.^3^

According to the state of sweat glands involvement, two major groups are distinguished: (1) Hypohidrotic or anhydrotic (Christ-Siemens-Touriane syndrome) in which sweat glands are either absent or significantly reduced in number; (2) Hydrotic (Cloustone syndrome) in which sweat glands are normal. Dentition and hair are involved similarly in both types but hereditary patterns of nails and sweat glands involvement are different. ^4, 5^ Hypohidrotic ectodermal dysplasia as the most common type seems to show an X-linked inheritance pattern with the gene mapping to Xq12-q13; therefore, males are more susceptible than females. Hydrotic type is inherited in an autosomal dominant pattern.^6^ In general, the skin of affected children is lightly pigmented and appears thin and almost transparent; surface blood vessels are easily visible. Other manifestations include fine sparse hair, reduced density of eyebrow and eyelash hair. When hair is present, it may be fragile, dry, and generally with unruly appearance as a result of poorly developed or absent sebaceous glands. Fingernails and toenails may also show faulty development and be small, thick or thin, brittle, discolored, cracked, and/or ridged.^4^

The pre-ocular skin may show fine wrinkling with hyper-pigmentation and midface hypoplasia is frequently observed resulting in protuberant lips. In cases where the salivary glands are hypoplastic or absent, varying degrees of xerostomia are expected.^7^ Affected individuals typically display heat intolerance because of reduced number of eccrine sweat glands. These glands may be either absent, reduced in number, or nonfunctioning (hypohydropic), which may result in elevated body temperature.^8^ Fever with unknown origin may lead to early diagnosis during infancy.

The teeth are markedly reduced in number (oligodontia or hypodontia) and often manifest abnormal development in shape which may appear tapered, conical or pointed in incisors. Molars might be observed in reduced size.^10^ The lack of tooth bud formation causes hypoplastic alveolar bone, leading to a reduced vertical dimension of occlusion. Therefore, an old-age appearance is common in affected individuals.

This article presents the early prosthetic rehabilitation for two children with hereditary ectodermal dysplasia associated with severe oligodontia in primary and mixed dentition.

## Case report

### Case 1

A 3-year-old boy was referred to the Department of Pediatric Dentistry, Shiraz University of Medical Sciences, representing lack of teeth as the chief complaint. According to familial history, a brother of the patient also suffered from oligodonatia and heat intolerance. Extraoral examination revealed dry skin, hypoplastic midface, prominent lips, and sparse fine hair. These findings matched typical features of anhidrotic ectodermal dysplasia (Figure 1a,b). The intraoral examination revealed complete edentulism in the lower jaw, four peg shaped anterior teeth in the upper jaw, thin alveolar ridge, reduced vertical bone height and loss of vestibular depth in the lower jaw (Figure 1c). The radiographic findings also confirmed the clinical diagnosis. Panoramic view revealed two unerupted molar teeth in the upper and two unerupted incisors in the lower jaw (Figure 1d).


Figure 1. Three-year old boy with anhidrotic ectodermal dysplasia. Frontal view (a); Profile view (b); Intraoral view (c); Panoramic radiograph (d).

**a:**
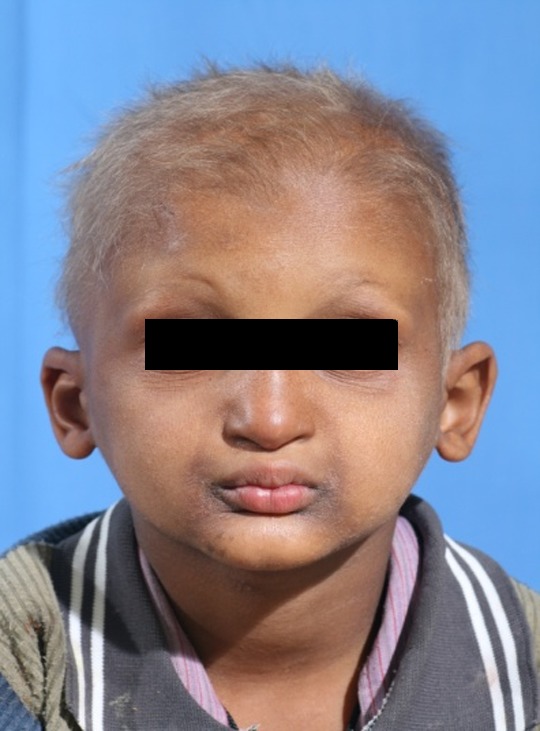

**b:**
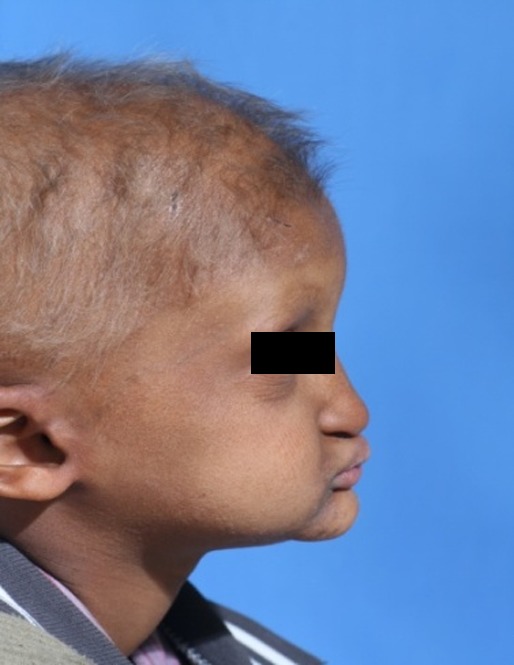

**c:**
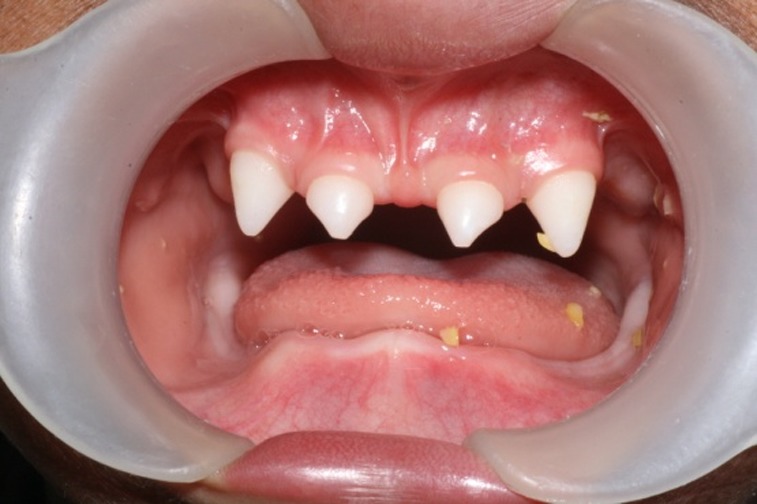

**d:**
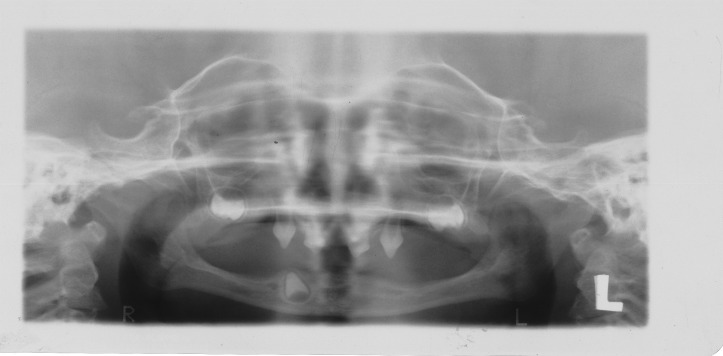

Treatment plan included making complete overdentures for the upper and lower jaws to improve appearance, function and speech. Due to small arch size, preliminary impression was made by modeling plastic as custom tray and zinc oxide impression material. The lower anterior teeth were left unmodified because of short crowns. When properly fitting custom tray was ready, final impression was made by elastomeric materials. Jaw relationship was recorded using temporary base and wax rim. The arranged teeth were verified in the mouth during the try-in appointment. After laboratory processing, the overdentures were delivered to the patient (Figure 2). In the delivery appointment, instructions were given to maintain proper oral hygiene. Continuous follow-ups every six months were planned for adjustment or replacement of old denture.


Figure 2. Maxillary and mandibular overdentures in place.

**a:**
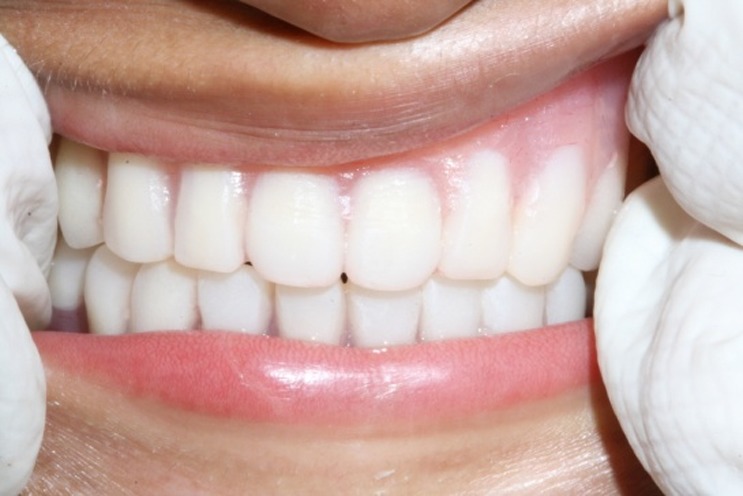

**b:**
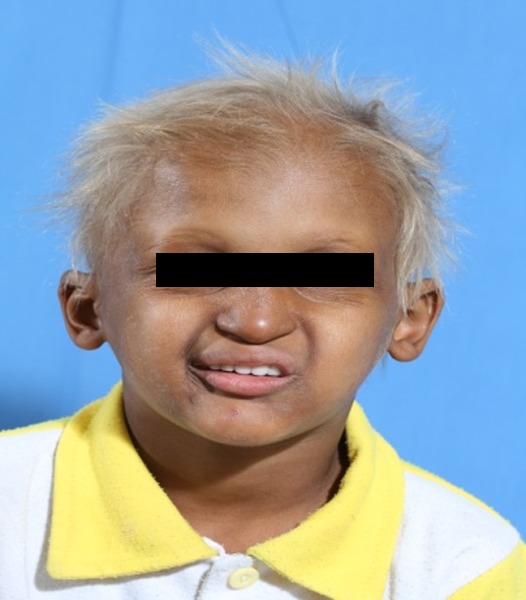

### Case 2

A 10-year-old girl was referred to the Department of Pediatric Dentistry, Shiraz University of Medical Sciences, representing oligodontia. Familial history revealed that close relatives including grandmother on the mother’s side and cousins were afflicted with the same condition. In extraoral examination, sparse fine hair, dry skin, reduced vertical height of facial lower third and prominent chin and lips were observed (Figure 3a). Intraoral examination revealed four primary molars, five permanent molars, three incisors, and unilateral maxillary contraction (Figure 3b). One permanent semi-erupted molar tooth and three unerupted premolars were found in radiographic view (Figure 3c). These findings matched typical features of anhidrotic ectodermal dysplasia.

In order to improve appearance and mastication, removable partial prosthesis was established as treatment plan. First, maxillary crossbite was corrected using Hawley appliance for six months. The two anterior peg-shaped teeth were restored by composite resin. In order to fabricate removable partial denture, two impressions were made with a hydrocolloidal material (alginate); first with stock tray and then with custom tray. Removable partial prostheses were fabricated in coordination with patient’s occlusion and wrought wire clasps were used as retainers (Figure 3d). Recalls were scheduled every 6 months to evaluate developing jaws and teeth eruption.


Figure 3. Ten-year old girl with anhidrotic ectodermal dysplasia. Profile view (a); Intraoral view (b); Panoramic radiograph (c); Maxillary and mandibular removable partial dentures in place (d).

**a:**
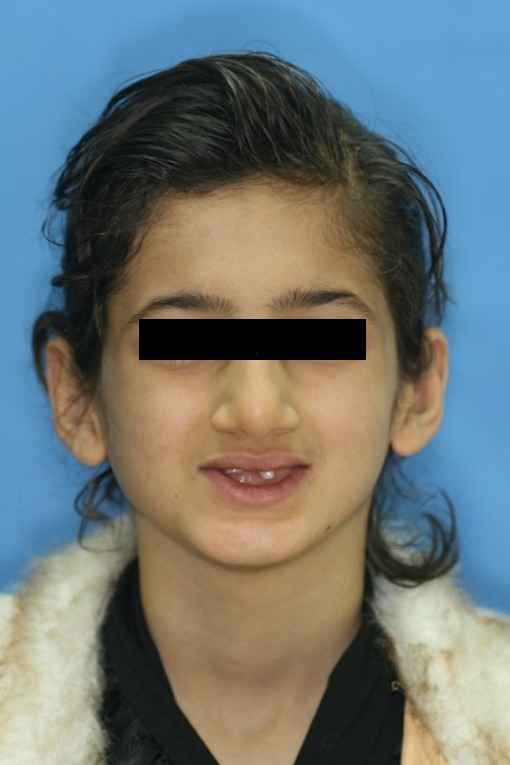

**b:**
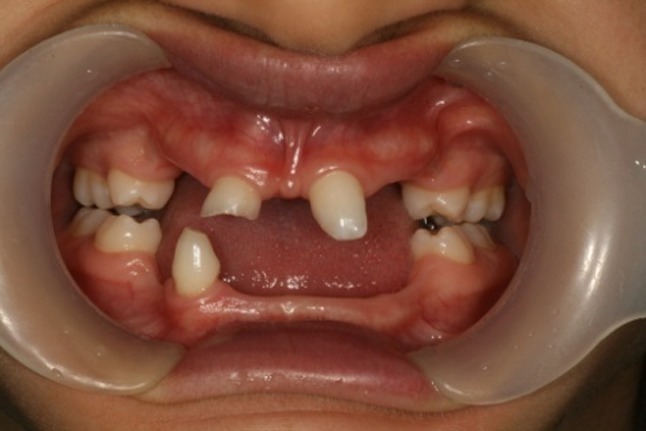

**c:**
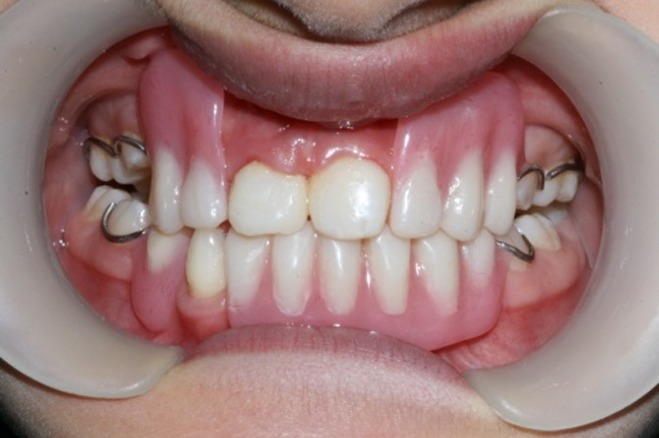

**d:**
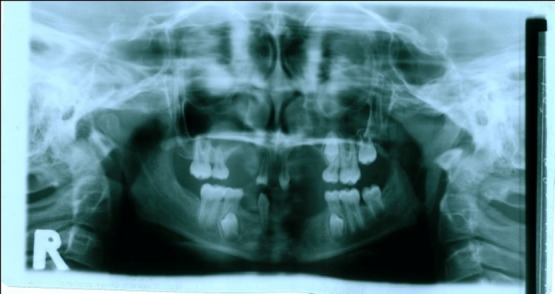

## Discussion

Oral rehabilitation of patients with ectodermal dysplasia is necessary to improve sagital and vertical skeletal relationships during craniofacial growth and development as well as esthetics, speech, and masticatory efficiency.^4^ The most common treatment plan is removable prosthesis. Implant-supported denture is also suggested as the ideal reconstruction modality for adolescents over 12 years. When implant therapy is indicated, the main problem is insufficient bone; if bone atrophy progresses in these already alveolar deficient patients, implant placement may not be possible without bone grafting.^8^ In addition to the psychological effects particularly in young children, implant surgery is accompanied by a higher risk of failure compared to that of more conservative prosthetic treatments.^7 , 11^ Early implant placement in a growing child may cause cosmetic problems because implants act similar to ankylosed teeth. Along with the craniofacial growth, implant over-structures may not be in occlusion with opposite teeth and even the adjacent teeth may tilt into the space. Thus implant supported prosthesis may be less favorable,^8 , 12^and therefore, the use of implants in young children should be considered carefully. Early prosthetic treatment is generally recommended from the age of 5 years. With regard to child cooperation, dentures can also be fabricated as early as 3 to 4 years of age.^13^ Positive effects include more self-confidence, facial esthetics, speech and masticatory function improvement.^13^

However, removable partial or complete dentures require regular adjustments and should be replaced when a decreased vertical dimension of occlusion and an abnormal mandibular posture are detected due to growth.^14^ Retention and stability for the prostheses are also difficult to obtain. In patients with ectodermal dysplasia, dryness of the oral mucosa and the under-developed maxillary tuberosities and alveolar ridges are problematic factors for resistance and stability of the dentures.^15^ When fabricating dentures for these patients, care should been taken to obtain a wider distribution of occlusal loads by extending the denture base as much as possible. Although the atypical conical anterior teeth may not be suitable for removable partial denture stability, they may be used as abutments for overdentures.^16 - 18^ In this situation, special attention must be paid to the impression making technique. The occlusion of removable partial denture should be in harmony with the patient’s occlusion. Since oligodontia or anodontia leads to atrophy of the alveolar ridges, reduced vertical dimention, prominent chin, and class III intermaxillary relationship, early prosthetic treatment should be performed as soon as possible. This treatment modality improves the patient’s quality of life and it can be regarded as an acceptable treatment modality for functional and esthetic rehabilitation.

## Conclusion

Management of clinical manifestations associated with ectodermal dysplasia presents a unique challenge for prosthodontists and pedodontists. Treatment of young edentulous patients with removable partial or complete denture is an acceptable, available and cost effective modality, which improves function, speech, esthetics and psychosocial condition. However, its long-term success depends on regular recall appointments and meticulous maintenance of oral and prosthetic hygiene.
